# Cryopreservation of Fish Spermatogonial Cells: The Future of Natural History Collections

**DOI:** 10.1038/s41598-018-24269-3

**Published:** 2018-04-18

**Authors:** Mary M. Hagedorn, Jonathan P. Daly, Virginia L. Carter, Kathleen S. Cole, Zeehan Jaafar, Claire V. A. Lager, Lynne R. Parenti

**Affiliations:** 1 Smithsonian Conservation Biology Institute, Front Royal, VA 22360 United States of America; 2 Hawaii Institute of Marine Biology, 46-007 Lilipuna Rd, Kaneohe, HI 96744 United States of America; 3 University of Hawai’i at Mānoa, 2538 McCarthy Mall, Edmondson Hall 216, Honolulu, HI 96822 United States of America; 4 Department of Biological Sciences, 14 Science Drive 4, National University of Singapore, Singapore, 117543 Singapore; 5 Department of Vertebrate Zoology, P.O. Box 37012, MRC 159, National Museum of Natural History, Smithsonian Institution, Washington, D.C. 20013-7012 United States of America

## Abstract

As global biodiversity declines, the value of biological collections increases. Cryopreserved diploid spermatogonial cells meet two goals: to yield high-quality molecular sequence data; and to regenerate new individuals, hence potentially countering species extinction. Cryopreserved spermatogonial cells that allow for such mitigative measures are not currently in natural history museum collections because there are no standard protocols to collect them. Vertebrate specimens, especially fishes, are traditionally formalin-fixed and alcohol-preserved which makes them ideal for morphological studies and as museum vouchers, but inadequate for molecular sequence data. Molecular studies of fishes routinely use tissues preserved in ethanol; yet tissues preserved in this way may yield degraded sequences over time. As an alternative to tissue fixation methods, we assessed and compared previously published cryopreservation methods by gating and counting fish testicular cells with flow cytometry to identify presumptive spermatogonia A-type cells. Here we describe a protocol to cryopreserve tissues that yields a high percentage of viable spermatogonial cells from the testes of *Asterropteryx semipunctata*, a marine goby. Material cryopreserved using this protocol represents the first frozen and post-thaw viable spermatogonial cells of fishes archived in a natural history museum to provide better quality material for re-derivation of species and DNA preservation and analysis.

## Introduction

The persistent decline in global biodiversity has renewed interest in the study of natural history of organisms and sparked debates on the collection and use of biological specimens^1,2^. Natural history collections are the foundation of a broad range of biological disciplines including systematics, ecology, functional morphology, reproductive biology, and conservation. Despite the differing goals of scientists in these fields, all agree that the value of natural history collections increases over time. These specimens are not only irreplaceable^1^, their potential uses evolve with technological advancements and new research questions^3^.

Natural history collections include whole small vertebrate specimens, especially fishes, traditionally fixed in formalin and subsequently transferred to alcohol for long-term storage. This preservation method is appropriate for the conventional use and long-term storage of these specimens, but yields poor quality tissue samples for molecular analyses^4^. The past two decades have seen a marked increase in the number of tissue or whole animal samples preserved in ethanol or frozen, in freezers or liquid nitrogen, and deposited in natural history collections. With the technology to easily capture and analyze molecular sequence data, ethanol-preserved and frozen tissue samples are now a ready source of data for phylogenetic systematic analyses. Substantial funds are committed to collect, curate, and store these materials. Yet sample materials may yield poor quality sequence data, especially after several years in storage^5^. Even with continued improvements in extraction protocols and methods to analyze large datasets, tissue degradation hinders large-scale generation of molecular sequence data. Next Generation sequencing technologies allow researchers to sequence whole genomes with dramatically reduced investments of time and money, but these too require larger quantities and higher quality DNA than that of traditional sequencing of PCR products using a few genetic markers. We present an alternative: the cryopreservation of diploid spermatogonial cells, living tissue that, if maintained properly, can yield better quality tissue and therefore yield more complete (reliable) DNA sequence data.

Cryopreserved spermatogonial cells might also be used to regenerate fish populations. Yet expectations that banked cells can save all fish species must be conservative due to the complexity of chromosomal sex determination in fishes. Fishes exhibit an astounding range of variation in the presence or absence of sex chromosomes (or for that matter, sex chromatin), sex-determining systems, polyploidy^6–8^ and even non-genetic adjustment of sexes related to water temperature^9^. For example, the transfer of XY spermatogonia A-type cells into different hosts will generate XY spermatids or XY ova, in separate individuals. The subsequent fertilization of these XY ova and XY sperm will give rise to offspring in these percentages: 25% female (XX), 50% male (XY) and 25% probably non-viable (YY). The next generation (F_2_) may be normal, but females, the source of the limiting gamete, will be less common in the first generation than males (1:2) in male heterogametic species. In male homogametic species, all offspring will be genotypic males. The chromosomal sex determination pattern for *A. semipunctata*, and for many other reef fish species, is unknown. Nevertheless, these samples are still extraordinarily valuable. Because testicular samples are frozen, yet alive, their genetic material remains largely unaltered over many years^10^. Long-term studies are beginning to assess the genetic stability of these new kinds of frozen collections^11^. For natural history collections, these frozen collections must pass the test of hundreds of years of usefulness, as have their dried, alcohol- and formalin-fixed counterparts.

Cryopreservation is one conservation technology with the potential to become central in natural history collections worldwide. For over 65 years, cryopreservation has been an important tool to preserve biological diversity^12^; the field of cryo-technology continues to advance as a practical and cutting-edge science. To cryopreserve a cell, water is extracted and subsequently replaced with a cryoprotectant or antifreeze material. The partially dehydrated cell can then withstand the extraordinary stress from exposure to ultra-cold temperatures (ca. −196 °C) in a state of suspended animation^13,14^. Cells that are correctly frozen and banked can remain viable for years without damage to DNA^14^, and thus provide a means to potentially safeguard all extant species and their genetic diversity. These ‘frozen banks’ allow: (1) large samples of preserved and protected gene pools to ‘seed’ shrinking populations, even to ‘resurrect’ extinct species; (2) easy and inexpensive transport of genetic materials among living populations, such as small populations in zoos; (3) access to quality biomaterials for scholarly research; (4) invaluable opportunities to maintain individual genomes of rare species; and, most important, (5) the capture and distribution of genetic diversity. Nevertheless, cryopreservation technology is perceived narrowly as the purview of fisheries biologists to provide sperm to fertilize commercially valuable species^15^ or of conservation biologists based in zoos to maintain living populations of charismatic species such as the Giant panda, the Black-footed ferret or large, predatory cats^16,17^. Despite the immense scientific value of cryopreservation, live cryopreserved cells are not maintained in natural history museums. These cells—and their functionally preserved, diploid/somatic DNA—are essential for the successful rederivation of species. The absence of these collections is due in part to the lack of established and standardized collection and preservation protocols for many wildlife cells. Yet, the cryopreservation technology for fish cells is burgeoning. Our goal is to make cryopreservation a more accessible tool for museum collections.

Recent discoveries in mammalian and other vertebrate lineages have highlighted the pluripotency of spermatogonial stem cell lines that display enormous transformational potential^18–20^. Transplanted spermatogonial stem cells of salmonid fishes differentiated into mature sperm and oocytes and produced offspring between both intra- and interspecies donors and sterile hosts^21^. Recently, cryopreserved and thawed testicular cells have been combined to produce both viable eggs and sperm^11,22,23^. These testicular cells will diversify and expand the resource options for the vast number of cell lines that need secure resource banking.

Spermatogonia of many fish species are relatively large (reported to be 12 to 16 µm in diameter), self-renewing, diploid, testicular cells present in the testes in varying numbers year-round^24^. The largest cells in the germ line, spermatogonia A-type divide via mitosis into two diploid daughter cells: spermatogonium A-type and spermatogonium B-type cells. Spermatogonia B-type cells are smaller (approximately 9 to 12 µm in diameter). Once surrounded by Sertoli cells, they become primary spermatocytes^25^. Primary spermatocytes are similar in size and shape to spermatogonia B-type cells and are also diploid. Spermatogenesis proceeds as the primary spermatocytes enter meiosis to ultimately form haploid sperm (1 to 3 µm in diameter)^24^.

Cryopreservation of spermatogonial testicular cells has the potential to save fish species worldwide because of spermatogonial capacity to regenerate organisms, a new tool to counter species extinctions. The work of some fish researchers in this area^21–23^ is not yet conservation-ready, as it focuses on transgenically modified fishes with fluorescently labeled VASA-positive spermatogonia A-type cells or non-breeding fish with testes dominated by spermatogonia A-type cells. If these testicular cryopreservation methods are to become common, they will have to be adaptable/usable for a wide range of fish species.

**Figure 1:**
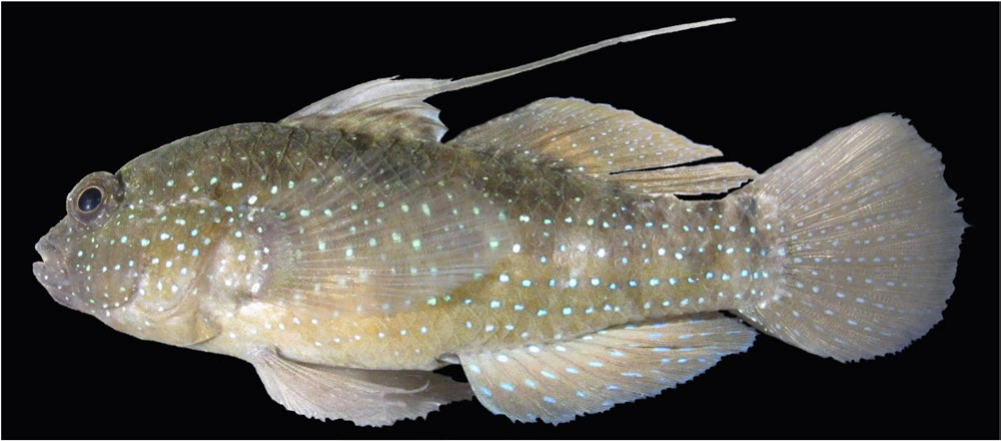
Fish species tested for spermatogonial cell cryopreservation: *Asterropteryx semipunctata*, USNM 421677, male, 37.4 mm SL.

Our goal is to develop and standardize spermatogonial cell cryopreservation protocols by testing and modifying previous testicular cryopreservation methods^21–23^. We chose the Indo-Pacific marine Starry goby, *Asterropteryx semipunctata* Rüppell 1830 (Fig. 1), as a model. This relatively small, common species attains a maximum adult size of less than 50 mm standard length. *Asterropteryx semipunctata* belongs to one of the largest families of bony fishes, the Gobiidae, which comprises an overwhelming abundance and biomass of the fish communities in estuaries, mangroves, and coral reef habitats^26^. Small cryptic reef fishes such as gobiids are ecologically important as they use various trophic pathways, particularly detritivory^27^. Moreover, it is estimated that fishes smaller than 50 mm SL, which includes the majority of reef-dwelling gobies, contribute over 25% of the total energy flow in coral reef communities^26^. Successful cryopreservation of the spermatogonial testicular cells of this model fish species will establish a method that may be extended to other fishes and vertebrates.

We first applied existing protocols as a springboard to build a field-hardy method to cryopreserve spermatogonial cells and thereby demonstrate that the collection and preservation of these tissues is practical and cost-effective. We then compared it to two other methods— one based on the research of Lee *et al*.^22,23^ and our modification of the method of Lee *et al*. We assessed our success in preserving viable spermatogonia post-thaw with fluorescence and flow cytometry, focusing on the larger cells in the testes (≥10 µm), thereby ensuring a population of the self-renewing spermatogonia A-type cells.

We then transferred the frozen material to the National Museum of Natural History, Smithsonian Institution (USNM), to begin the first frozen diploid, spermatogonial cell collection for fishes in a natural history museum. Through these methods, we encourage the collection and long-term storage of cryopreserved tissues of a broad range of taxa in natural history museums worldwide.

## Methods

### Collection Methods and Animal Care

We collected wild *A. semipunctata* from two field sites on Coconut Island in Kaneohe Bay, O’ahu, Hawaii (21°26′38.5″N, 157°47′47.2″W): sandy reef flats off the northeast (Sandflat Fore Reef Beach) and northwest (near the Lanai Suites) coast of the island. Fish were identified visually using snorkeling gear, captured using small handnets and transported within 5 min to the laboratory at the Hawaii Institute of Marine Biology (HIMB). Maintenance and handling of live fishes met the animal care standards of the National Institutes of Health. Full details of the study approval are listed with the Smithsonian Conservation Biology Institute (SCBI) IACUC (approval ID #12-32), USNM IACUC (approvals ID #2013-06; #2017-01) and the HIMB, University of Hawaii IACUC (protocol ID# 12-1491). Fishes were collected under permit SAP-2013-47 and SAP-2018-35 from the Department of Land and Natural Resources, Hawaii.

### Specimen Preparation

Live fish were immersed in a 0.01% solution of buffered MS-222 until gill movement ceased and there was no response to mechanical stimulation (~5 min). Individuals were rinsed with clean water and placed dorsal surface down on a damp sponge or paper towel. Information on each individual, including sex (based on urogenital papilla morphology^28^) and size (Standard Length, the straight-line distance from the tip of the snout to the base of the caudal fin, mm) was recorded. A specimen was identified as a juvenile if we could not determine sex by visual inspection. We surgically removed testes and accessory gonadal structures (see below) from adult male specimens, following a standard protocol^29^. Surgery was performed under a stereomicroscope (Wild M5). An incision was made with micro-dissecting scissors to expose the abdominal cavity. Paired testicular lobes or paired testicular lobes and associated paired accessory gonadal structures, the latter a male reproductive structure diagnostic of gobioid fishes^30,31^, were removed and placed into chilled (0 °C) Eagle’s Medium with 5% fetal bovine serum (FBS) and 2 mM L-glutamine. Following surgery, small samples (ca. 2 mm × 2 mm) of myomeric musculature from the abdominal wall or the pectoral fin, or the accessory organs, were removed from select specimens, placed into 95% ethanol and stored at 22–23 °C.

**Figure 2:**
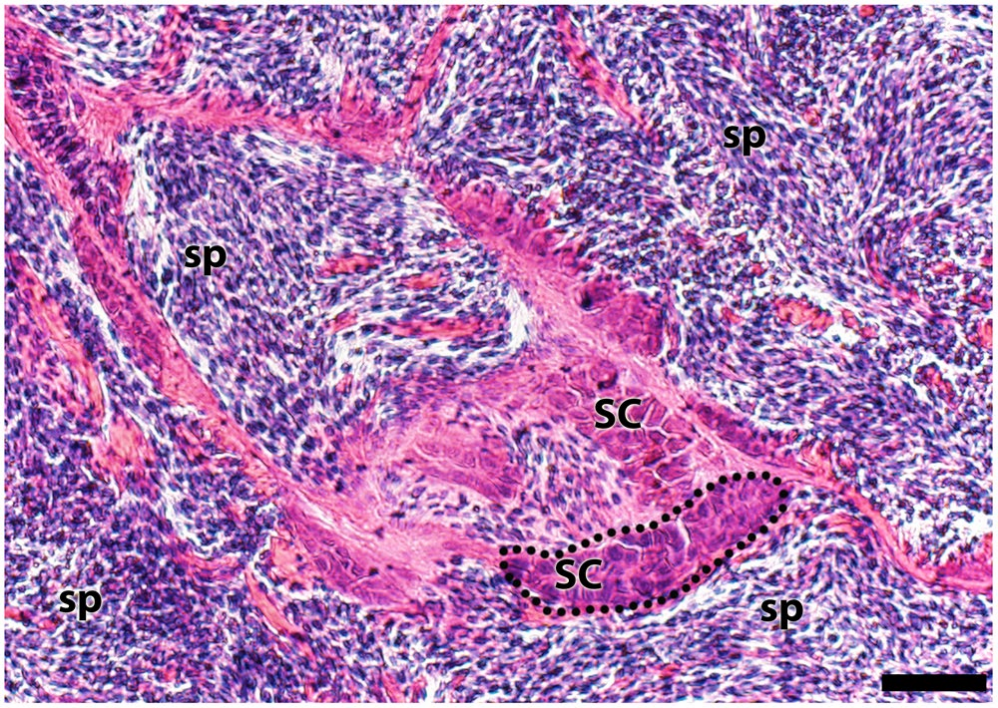
Histological section through the testis of *Asterropteryx semipunctata* (USNM 410667), male, 29.9 mm SL. The testis is an unrestricted lobular type with spermatocysts (SC); the lobules are full of spermatozoa (sp). Dotted lines approximate the border of one spermatocyst. Bar = 20 µm.

To assess reproductive state, we examined the testes of one male via histology. A testis was dissected out of an adult male, *A. semipunctata* (USNM 410667, 29.9 mm SL), collected on 25 April 2015. The specimen had been fixed in 10% formalin and preserved in 75% ethanol. The testis was embedded in Paraplast Xtra and sectioned at 6 µm using a Leica 2255 automated microtome. Sections were stained with Hematoxylin and Eosin (H&E). Slides were examined with a Leitz light microscope and photographed using an Olympus BX63 microscope equipped with a DP-80 digital camera and using Olympus cellSens version 1.13 imaging software. All histological slides are maintained in the Division of Fishes, USNM. The *A. semipunctata* testis is of the unrestricted lobular type as described previously^32^, containing spermatocysts along the lobules (Fig. 2). The lumen of the lobules was full of spermatozoa, which indicates that this species was reproductively active during the period of our experiments.

We collected 45 voucher specimens plus ethanol-fixed tissue samples of *A. semipunctata* (USNM 421647–421691) from the two HIMB localities, in 2013. Select tissue samples were sequenced to identify the species-specific DNA Barcode in order to test the hypothesis that our specimens represented a single species (see Supplemental Information).

In 2015, we collected 10 adult males from which we cryopreserved testicular spermatogonial cells for use in Experiment 1; the formalin-fixed voucher specimens were catalogued as USNM 407874-407880, 410554, 410559 and 410570. Cell suspension samples from 10 adult males collected in 2017 (vouchers USNM 442176, 442179, 442182-442188, and 442192) were banked from Experiment 2. Testicular cells from nine adult males collected in 2017 (vouchers USNM 442200, 442205, 442227, 442228, 441795, and 441797–441800) were banked from Experiment 3. Whole testes from eight more males (vouchers USNM 441802–441809) collected in 2017 were cryopreserved directly in CPA 1 (see below) and banked. An additional 49 males and females from our 2015 and 2017 collections were fixed in formalin and transferred to ethanol as whole specimen vouchers: USNM 410667, 435346, 442149, 442151-442153, 442158–442161, 441978–441980, 441931, and 441933, and Bernice P. Bishop Museum (BPBM) 41336 (ex. USNM 442149). Cryopreserved testicular samples were loaded into a liquid nitrogen dry shipper and transported from Hawaii to the National Museum of Natural History (NMNH) Biorepository in Suitland, MD, USA. Specimen vouchers were deposited in the NMNH National Collection of Fishes. Data for all specimens of *A. semipunctata* collected from 2013 through 2017 are available from the online catalog of the Division of Fishes: http://collections.mnh.si.edu/search/fishes/.

### Experiment 1

**Figure 3:**
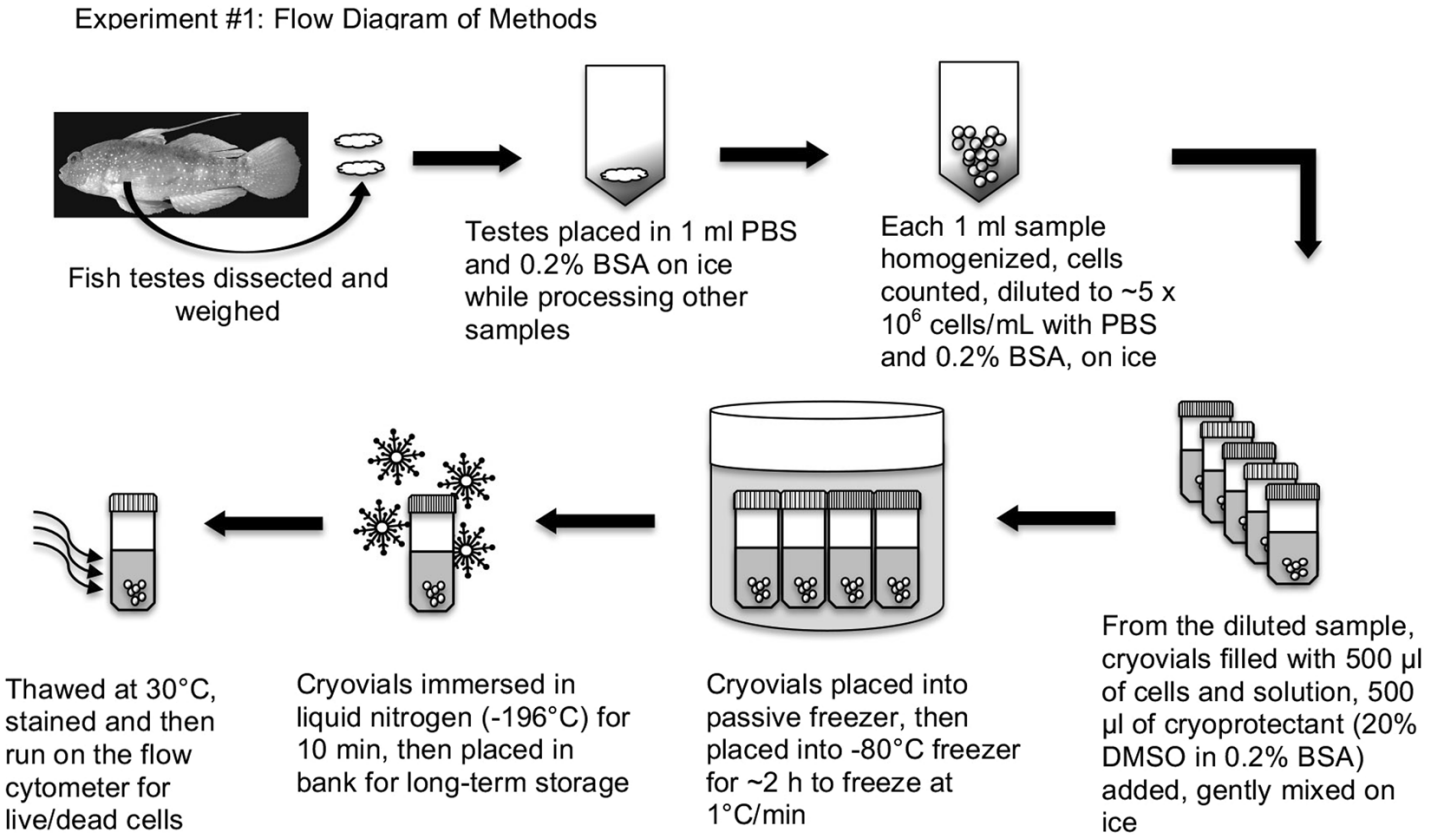
Flow diagram of Experiment #1 demonstrating the protocol for the cryopreservation of fish testicular cells as described in Experiment 1.

A preliminary study was undertaken on 10 adult males to develop methods for the dissociation of testicular cells, assess cell viability, cryopreserve the cells and compare two passive freezing devices for cryopreservation of dissociated cells. While the gross dissections proceeded, one lobe of the testes or a lobe and the associated accessory gonadal structure(s) of *A. semipunctata* were held in 1 ml of PBS and 0.2% BSA in an Eppendorf microfuge vial on ice. Once dissections were complete, the contents of each Eppendorf were transferred individually into a glass homogenizer and crushed until all cellular clumps were eliminated (~20 strokes; Fig. 3). The homogenizer was cleaned in freshwater, rinsed in 70% ethanol and dried between individual cell homogenizations. The cellular concentration of each sample was determined with a hemocytometer, diluted to ~5 × 10^6^ cells/ml with chilled PBS and 0.2% BSA, and viability determined prior to cryopreservation (see description of viability assay below). Then, 500 µl aliquots of the diluted dissociated cells were placed into non-sterile 2-ml labelled cryovials. This was then mixed with 500 µl of a 20% cryoprotectant solution (dimethyl sulfoxide, DMSO, made up by vol/vol with PBS), held at 22 to 23 °C for 10 min to help equilibrate the cryoprotectant into the testicular cells for a total of up to 10 cryovials per fish sampled. Cryovials were placed into one of two passive freezers, one with alcohol surrounding the cryovials (Mr. Frosty^TM^, Fisher Scientific) the other a thermo-conductive alloy and insulating materials surrounding the cryovials (CoolCell®, BioCision). Both passive freezers with cryovials were placed into a −80 °C freezer which cooled the cryovials at a rate of 0.5 to 1.0 °C/min down to −80 °C. After reaching −80 °C (~4 to 8 h), the cryovials were removed from the freezer and immersed in liquid nitrogen in which they reached −196 °C by 2 min.

To assess viability, samples were stained with propidium iodide (PI; 12 µM) and incubated in the dark at room temperature then analyzed on a BD Accuri flow cytometer^®^ (Becton Dickinson, Franklin Lakes, NJ, USA). For flow cytometry, 10,000 events (cells) were analyzed per sample and PI-positive cells (i.e. membrane-compromised cells) were identified. Along with the fresh and cryopreserved samples, two controls were included in all experiments to help identify: live and dead cells; (1) unstained, untreated cells; and (2) cells destroyed by 3 cycles of freeze-thawing followed by staining.

### Experiment 2

Testes from each male fish (n = 10) were dissected and placed individually into Eppendorf tubes containing PBS with 0.2% BSA on ice until all dissections were complete. Individual testes were homogenized as described in Experiment 1 and an aliquot was removed for analysis by flow cytometry to determine viability and concentration of the freshly isolated sample. The remaining sample was adjusted to ~5 × 10^6^ cells/ml with chilled PBS and 0.2% BSA then cryopreserved in 2.0-mL cryovials using a CoolCell® passive freezing device as described in Experiment 1. Cryopreserved samples were removed individually from storage with forceps, held in the air for three seconds to allow evaporation of excess liquid nitrogen, and then transferred into a water bath at 30 °C. The sample tube was swirled gently in the water bath to promote uniform warming, and the sample was completely thawed after 120 seconds. The sample and solution was then diluted 1:1 with PBS containing 0.2% BSA over a 5-minute period before fluorescent staining for viability.

**Figure 4:**
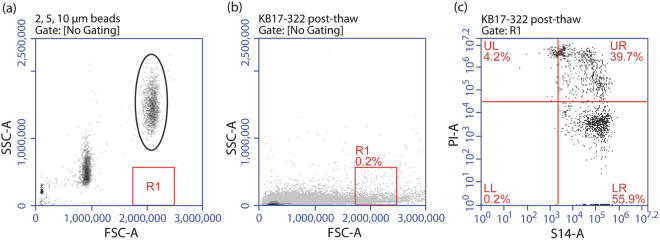
Flow cytometry scatter plots demonstrate the distribution of cells from homogenized testes of *Asterropteryx semipunctata* and the gating to separate cell populations. (**a**) Size calibration beads (2, 5, and 10 µm) were viewed on a forward scatter (FSC-A) vs. side scatter (SSC-A) plot. A gate (R1) was set below the population of 10 µm beads (oval) to help identify large testicular cells. (**b**) Testicular homogenates were viewed on FSC vs. SSC plots and events that fell within the R1 gate were viewed. (**c**) Fluorescent-stained cells within the R1 gate were displayed on SYBR-14 (S14-A, x-axis) vs. propidium iodide (PI-A, y-axis) plots to determine the number and proportion of cells with intact plasma membranes. These appeared in the lower right (LR) quadrant.

Viability of fresh and cryopreserved cells was assessed using the SYBR 14/PI membrane integrity assay (Molecular Probes, Eugene, OR, USA). Aliquots of 250 µL were stained with SYBR 14 (100 nM) and PI (12 µM) in Eppendorf tubes, and incubated in the dark at room temperature for ten minutes prior to flow cytometry. Samples (10 µL of sample at a flow rate of 35 µL min^−1^**)** were analyzed using a BD Accuri C6 Flow Cytometer^®^. Events were viewed on forward scatter (FSC) vs. side scatter (SSC) plots using CFlow^®^ software to allow separation of events based on cell size. This flow cytometer is able to measure the volume of sample collected, which allows calculation of sample event concentrations without the addition of counting beads. Gated events were viewed on a scatter plot showing SYBR 14 vs. PI to distinguish viable cells with intact membranes (i.e., stained with SYBR 14 alone) from membrane-compromised cells (stained with both SYBR 14 and PI or PI alone; Fig. 4). Samples were also viewed under a fluorescence microscope (Olympus BX41) to confirm fluorescent staining and cell sizes. Gating to separate the larger potential spermatogonia from the smaller sperm cells and debris was based on FSC using 10-µm polystyrene beads (SPHERO™ Particle Size Standard Kit, Spherotech, Lake Forest IL, USA) to identify events in this size range.

### Experiment 3

**Figure 5:**
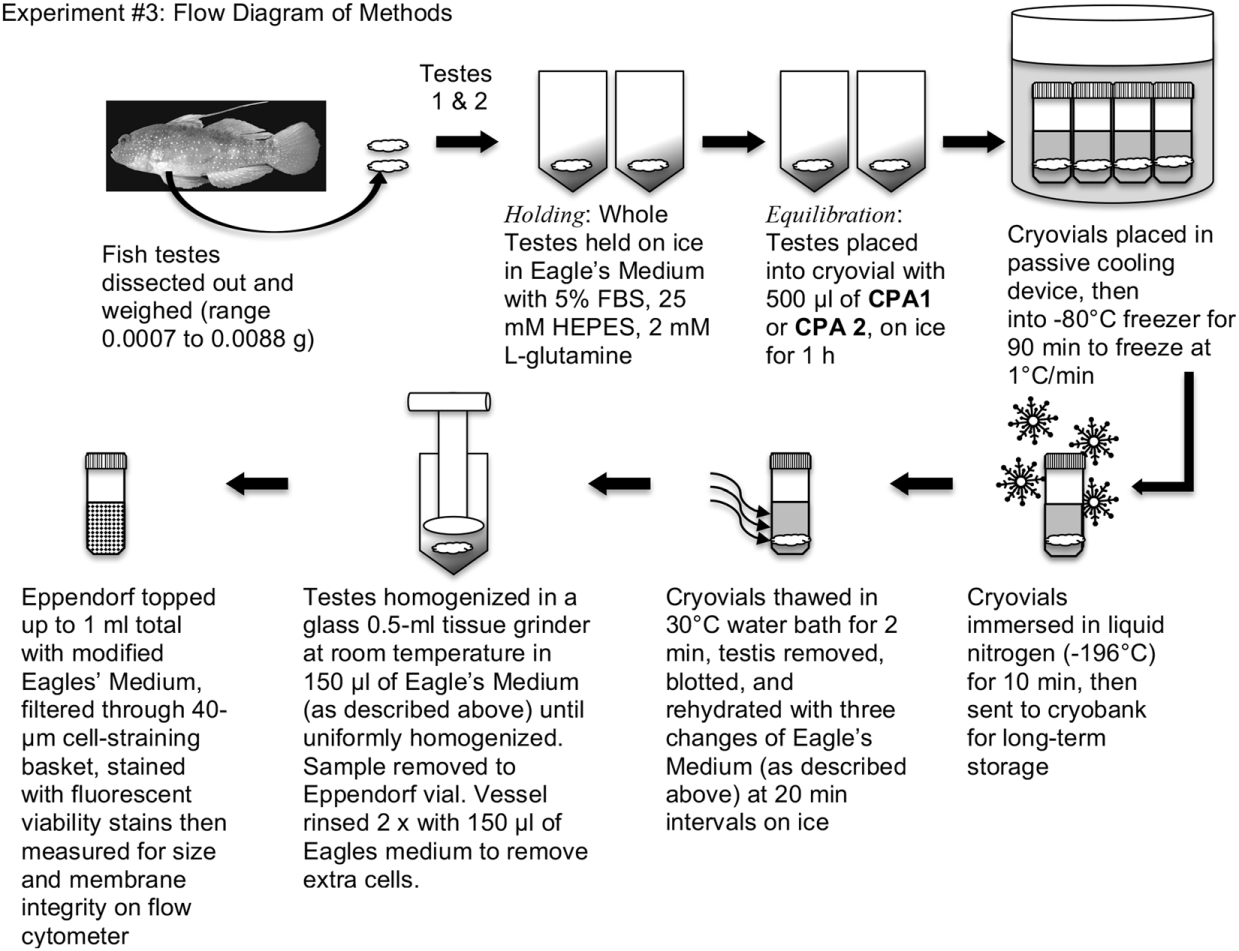
Flow diagram demonstrating the protocol for the cryopreservation of fish testicular cells as described in Experiment 3. The chemical composition of CPA 1 and CPA 2 are defined in Methods.

Dissections were performed as described above on nine adult males. Dissected testes were held individually in Eppendorf tubes containing Eagle’s medium supplemented with 5% FBS, 25 mM HEPES, and 2 mM L-glutamine, on ice. Two cryoprotectant solutions, designated CPA 1 and CPA 2, were used to cryopreserve whole testes. These are defined as CPA 1 (1.3 M dimethyl sulfoxide, 0.1 M trehalose, 1.5% BSA in 35.2% of Base medium (55.27 mM HEPES, 375.48 mM NaCl, 7,28 mM KCI, 23.1 mM KH_2_PO_4_, 3.82 mM Na_2_HPO_4_, 3.64 sodium pyruvate, 2.6 mM CaCl_2_r2H_2_O, 1.4 mM MgCl_2_r6H_2_O), pH 7.8 in deionized water) and CPA 2 (1.3 M dimethyl sulfoxide, 0.1 M trehalose in the Eagles’ Medium with 5% FBS, 25 mM HEPES, 2 mM L-glutamine). When all dissections were complete, paired testes from each fish were divided between the two CPA treatments, with one testis lobe from each fish placed into a cryovial containing 0.5 mL of CPA 1, and the alternate testis lobe placed into a cryovial containing 0.5 mL of CPA 2 (Fig. 5). Cryoprotectant 2 was designed to be a relatively simple cryodiluent and consisted of supplemented Eagle’s medium with 1.3 M DMSO and 0.1 M trehalose, which were equivalent to the cryoprotectant levels in CPA 1. Samples were equilibrated on ice for 60 min then cooled to −80 °C in a CoolCell® and stored in liquid nitrogen as described in Experiment 2.

Samples were thawed in a water bath at 30 °C. Each testis lobe was blotted to remove the CPA and rehydrated with three changes of supplemented Eagle’s medium for 20 minutes per change (60 minutes total). After rehydration, the testis lobes were blotted dry, transferred individually to a 0.5-mL glass tissue grinder with 150 µL of supplemented Eagle’s medium, and homogenized. The homogenate was removed to an Eppendorf tube and the tissue grinder rinsed with two changes to 150 µL of supplemented Eagle’s medium to collect any remaining cells. The sample was topped up to approximately 1 mL in the Eppendorf tube then filtered through a 40-µm cell strainer to remove any remaining pieces of tissue. After each testis was homogenized, the tissue grinder was filled with a 10% bleach solution and allowed to sit for 10 min to eradicate any remaining genetic material. The tissue grinder was then rinsed with deionized water and dried before the next testis was processed. Samples were analyzed with fluorescent staining and flow cytometry, using the protocol described in the previous experiment, to estimate the concentration of intact total, small and large cells in each CPA treatment.

### Statistical Analyses

Due to variation in standard length and testis size among males, cell concentrations were normalized against testis weight prior to statistical analyses. All statistical evaluations were performed using Graphpad Prism 5.0 (San Diego, CA) and Microsoft Excel (version 2007). Differences in the means were analyzed with General Linear Models which were fit using either independent or dependent groups, according to the hypotheses being tested, sample sizes and observational periods. Prior to statistical analyses, all percentage data were log-transformed. Results among pairs were evaluated with a two-tailed paired Student’s T-test. A level of *p* < 0.05 was considered significant, and all data were expressed in mean ± SEM.

## Results

### Experiment 1: Comparison of two passive freezing devices for cryopreservation of dissociated testicular cells

A preliminary study was undertaken to develop methods for the dissociation of testicular cells, assess cell viability, cryopreserve the cells and compare two passive freezing devices for cryopreservation of dissociated cells. Viability was determined in post-thaw samples (one cryo-sample per fish) using fluorescent live/dead staining. Each fish produced nine to10 cryovials of 1 ml of testicular cells. One cryo-sample was tested for post-thaw viability to determine success of the cryopreservation process using previous live/dead staining methods on a flow cytometer.

Homogenization of testes in a glass tissue grinder was found to be an effective means of isolating testicular cells in *A. semipunctata*, and the use of passive freezing devices for cryopreservation of dissociated cells resulted in live cells post-thaw. Samples cryopreserved in the CoolCell® had a slightly higher post-thaw viability (57.8 ± 2.8% SEM) than samples cryopreserved in the Mr. Frosty^TM^ system (55.9 ± 2.3% SEM), but this difference was not significant, so both systems are considered suitable for this type of tissue collection and preservation (*P* > 0.05, T-test). The CoolCell® was chosen for subsequent experiments as it was easier to use at low temperatures and did not require the addition of alcohol. There was little difference between these two passive freezers: there appeared to be an equal loss of cells in the freezing process of both, which we documented more fully in experiment 2.

### Experiment 2: Influence of dissociation/ nondissociation on the survival of cells during cryopreservation and thawing

**Figure 6:**
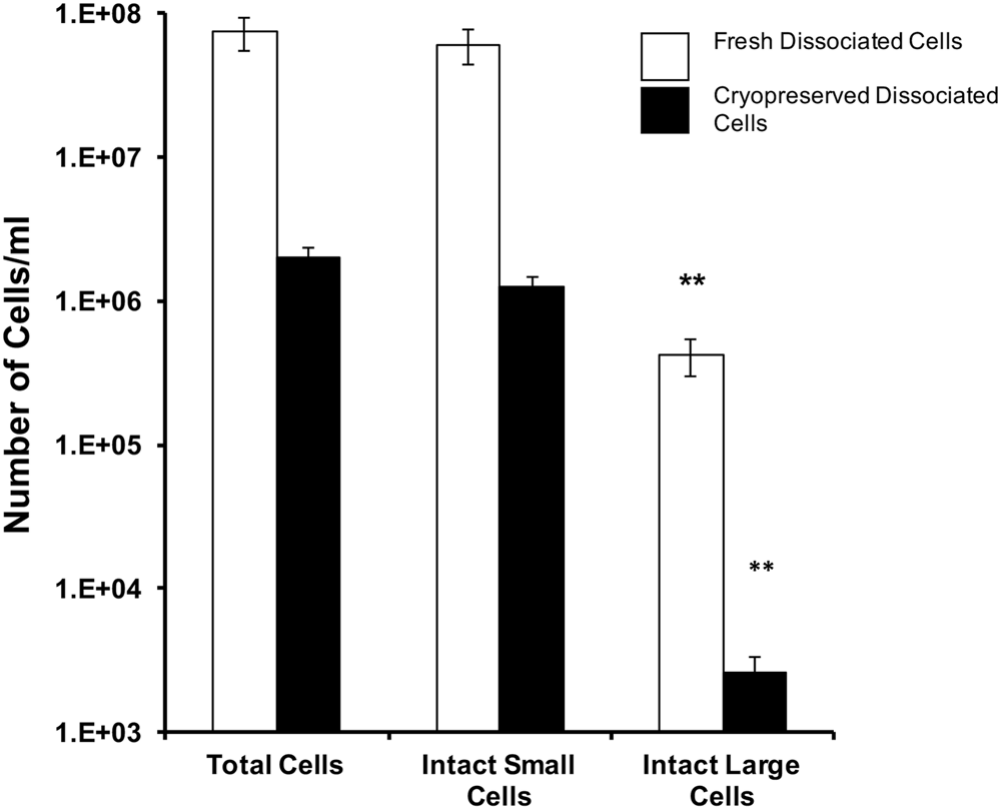
The cell concentration of dissociated fresh testicular cells versus dissociated cryopreserved cells was compared. Cell concentrations were measured in the fresh and the post-thaw cell preparations. The cryopreservation process caused an overall loss of cells compared to fresh (*P* < 0.05, F = 6.9), particularly in the large cells (*P* > 0.05). Bars with ** are significantly different from the intact cell numbers at *P* < 0.05, using a Dunnett’s post-test.

We sought to assess the concentration and proportion of viable dissociated testicular cells immediately after testicular homogenization and following cryopreservation using the methods developed in Experiment 1. Although viable cells were obtained following cryopreservation of dissociated cells in Experiment 1, it was not clear how these post-thaw viability results compared to freshly isolated testicular cells. Moreover, we had a concern that there was cellular loss during the dissociation, freezing, and thawing process and only this comparison could resolve that issue. Therefore, Experiment 2 examined both concentration and proportion of viable cells for possible pre-cryopreservation and post-thaw differences, to gain a better understanding of the effectiveness of the dissociated-cell cryopreservation technique (Fig. 6).

The creation of a gate to identify events corresponding to cells of approximately 10 µm in size enabled separation of large-sized spermatogonial cells (referred to as ‘large cells’) from the rest of the events on the scatter plot, which consisted mostly of smaller-sized spermatogonial cells (referred to as ‘small cells’). The cell concentration (cells/mL) of small cells, large cells, and total cells was quantified in fresh and post-thaw treatments (Fig. 6). Specifically within all the testes measured, the fresh treatments had the same number of mean intact total cells (7.4 ± 2.0 × 10^7^ cells/mL) and mean intact small cells (6.0 ± 1.7 × 10^7^ cells/mL) (*P* > 0.05), but fewer mean intact large cells (4.2 ± 1.2 × 10^5^ cells/mL) (*P* < 0.05, F = 8.37). The testes of all the fish were composed largely of spermatozoa at this time of year.

Once the cells were cryopreserved using the dissociated cell cryopreservation method, a tenfold reduction in cell concentration was noted for the intact total (2.0 ± 0.4 × 10^6^ cells/mL) and intact small (1.3 ± 0.2 × 10^5^ cells/mL) cell concentrations, and a 100-fold reduction in the large cell concentration (2.6. ± 0.7 × 10^3^ cells/mL) compared to the fresh samples. None of these means from the cryopreserved categories were different (*P* > 0.05), perhaps because of the large variation in the samples. Nevertheless, these losses in cell concentrations, particularly the large cell population, represented a considerable decrease in the potential utility of samples cryopreserved by this method.

### Experiment 3: Cryopreservation Success of Intact Testes Comparing Two Cryoprotectant solutions

**Figure 7:**
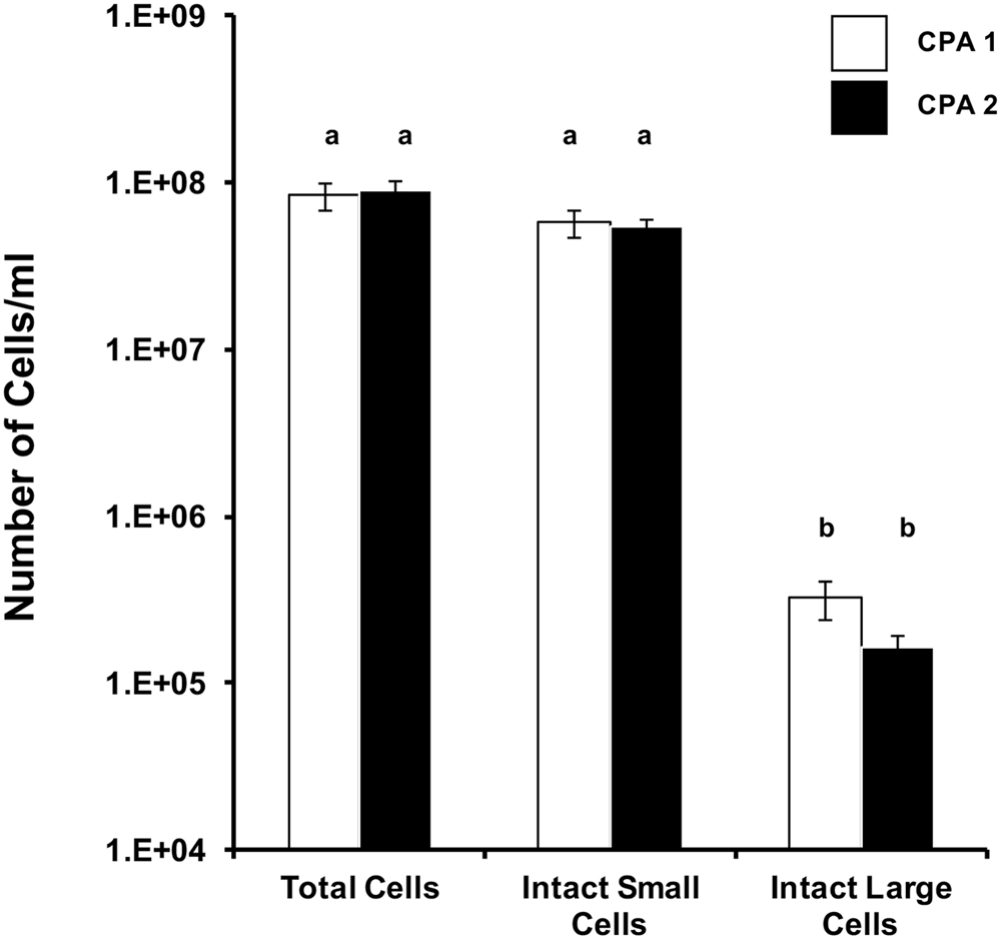
The cell concentration of cryopreserved whole testes, then dissociated post-thaw. Two cryoprotectant solutions were tested. Both cryoprotectant solutions cryopreserved the small and large cells equally well (*P* > 0.05, F = 21.9). Bars with different small letters are significantly different from the intact cell numbers at *P* < 0.05, using a Dunnett’s post-test. The chemical composition of CPA 1 and CPA 2 are defined in Methods.

Due to a reduction in cell concentration following cryopreservation of dissociated cells, we cryopreserved intact testes, comparing the methods established by Lee *et al*.^16,17^ to a simplified method for whole testis cryopreservation. This was designed to make testicular cryopreservation more accessible to users in the field or with minimal laboratory facilities, by utilizing a readily available medium to produce a simplified cryopreservation diluent. Two cryopreservation methods were tested towards creating a standard medium. The first was that of Lee *et al*.^16,17^ (CPA 1), and the second was a comparable method using a simplified cryoprotectant solution (CPA 2; Fig. 7). We were motivated to compare them to identify a simpler cryoprotectant solution that could be purchased readily and thus be widely available, a factor that would increase uniformity across field collections.

The extensive post-thaw cellular loss observed in the cryopreserved dissociated testicular cells was not observed in either of the whole testis cryopreservation methods tested. The total and small cell concentrations for CPA 1 and CPA 2 were virtually identical (Fig. 7). The big difference between these two cryoprotectant solutions was demonstrated in the large cell categories. The CPA 1 treatments produced 3.2 ± 0.9 × 10^5^ cells/mL while CPA 2 produced half that number of large cells (1.6 ± 0.3 × 10^5^ cells/mL), but this difference was not significant (*P* > 0.05), perhaps again because of the variability in the size, weight and cell numbers observed in the testes.

**Figure 8:**
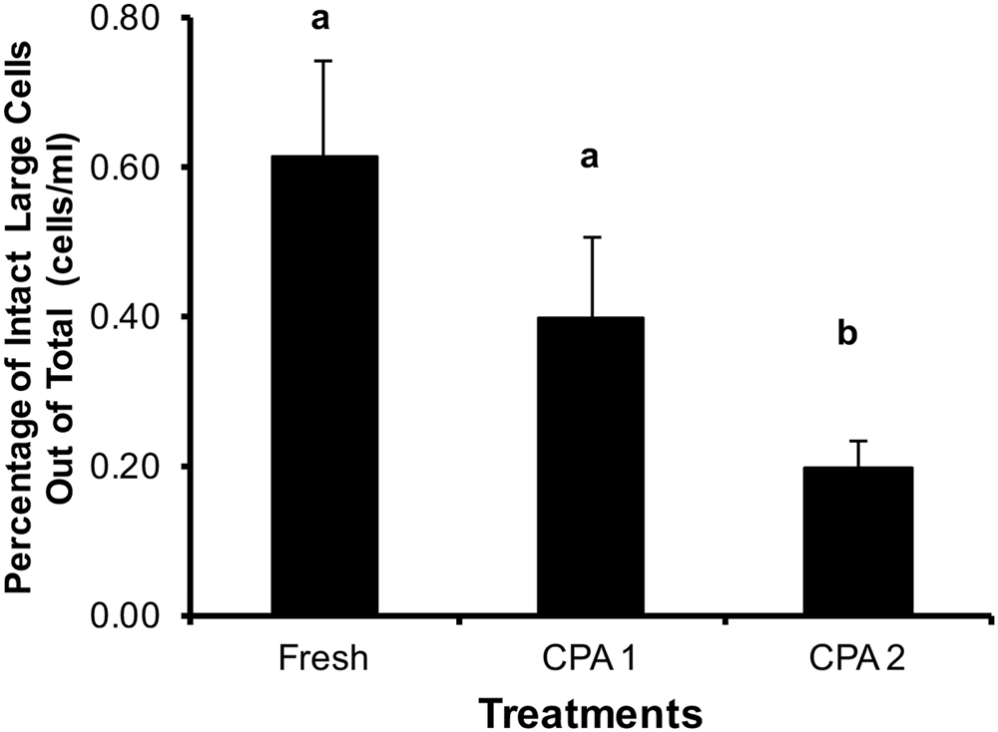
Percentage of intact large cells in various treatments. Bars with different small letters are significantly different at *P* < 0.05 (F = 4.9) treatments, using a Dunnett’s post-test. The chemical composition of CPA 1 and CPA 2 are defined in Methods.

A final comparison of these data examined what percentage of large cells was found in a total sample (cells/ml), and which cryopreservation method maintained the highest percentage of large cells (Fig. 8). We recorded approximately 0.62% large cells in a fresh ml of testicular cells, which is slightly higher than the proportion of large cells present post-thaw using CPA 1 (0.40%). Of note, this percentage was not different from the fresh (*P* > 0.05). However, the simplified cryoprotectant solution based on Modified Eagle’s Medium (CPA 2) was different producing only 0.19% large cells/ml (*P* < 0.05, F = 4.94). This indicated that although CPA 2 produced live spermatogonial cells post-thaw, it was not as good as CPA 1, which had the same level of live cells post-thaw as did the fresh control.

## Discussion

We present a robust protocol to cryopreserve spermatogonial cells from a marine goby, *A. semipunctata*, building on the successful cryopreservation protocols of many previous studies^11,22,23,33^. The cryopreserved material of *A. semipunctata* is, to the best of our knowledge, the first frozen spermatogonial cell collection of fishes in any natural history museum. It is also the first marine model to be cryopreserved and retained for long-term storage.

High-quality maintenance of living biological materials and their data requires a long-term commitment of personnel, supplies, and facilities. The collections management issues posed by frozen tissues are challenging and parallel those for traditional collections: (1) amount of material and sampling protocols to ensure diversity; (2) costs of continuous maintenance; (3) complexity of storing specimens that host symbiotic organisms or gut material; (4) database design; and (5) accessibility for researchers and collection managers. Like many standard collections, the majority of costs of cryo-collections lie in collecting, securing, accessioning, and cataloging the material. Afterwards, maintenance is a tractable operating cost. Most important, collections must remain viable, usable, and safe in perpetuity. The alternative we present here is the cryopreservation of diploid spermatogonial cells, living tissue that, if maintained properly, can yield the best and complete sequence data of both RNA and DNA.

One of our chief goals was to have a field-friendly protocol so that researchers could make these collections along with standard formalin-fixed vouchers. The simplified cryopreservation method presented here requires minimal laboratory facilities or reagents, and would be relatively simple to incorporate into existing collection procedures for museum specimens. As in other studies^33^, we tested the efficacy of dissociated versus whole testes for cryopreservation and found that the whole testes cryopreservation was superior at maintaining the integrity and viability of the large spermatogonial cells. The advantage of dissociated cells is that there can be many vials from the same individual in the bank to be used for a wide variety of purposes from genetic to conservation studies. Because of the unknown future uses of natural history museum collections, we banked both intact testes and dissociated cells. A disadvantage of the dissociated cells was the observed loss of large cells perhaps due to sub-lethal damage caused by homogenization that made the cells more susceptible to cryo-injury. In studies with freshwater fishes^33^, no difference was observed between the survival post-thaw of large cells cryopreserved as either whole testes or dissociated cells.

Another goal was to preserve the testicular cells of fish in the field regardless of their reproductive stage. Our species, *A*. *semipunctata*, spawns year-round with a peak during May through July^23^, therefore it was reproductively active during the period of our experiments. This increased the percentage of sperm and reduced the overall percentage of large spermatogonial cells in the testes, constraining our analysis of post-thaw large cells. Previous studies have used genetic constructs with Green Florescent Protein (GFP) to mark their viable spermatogonia cells^22,23^ post-thaw, used Percoll gradients to separate cells^34^ or increased their overall numbers of large cells in the testes by using immature males^33^. Some freshwater fishes showed cross-reactivity in VASA antibody staining of their spermatogonial cells, but *A*. *semipunctata* did not show any cross-reactivity (data not shown). Therefore, we used gating to select cells (~10 µm), but this grouping could include other large clusters of cells, such as spermatogonia B-type, and smaller cells, such as primary spermatocytes.

### Long Term Impacts/Projections

The cryopreservation method we endorse, as described in Experiment 3, is cost effective, practical, and easy to execute in the lab or the field. Reagents may be prepared ahead of time and frozen and cryopreserved testes transported back to the laboratory or museum in a standard liquid nitrogen shipper. A standard, commercially available cryo-solution would be of major benefit to future field collections worldwide, but it must perform as well as those described previously^22^.

Our research is part of the Pan-Smithsonian Cryo-Initiative (PSCI), a broad effort to oversee all frozen biological collections of the Smithsonian Institution. The PSCI includes over one million frozen tissue samples distributed across the Smithsonian, with over one-quarter of a million of those frozen samples maintained in mechanical freezers at −80 °C with generator back-up and nitrogen tanks at −196 °C at the National Museum of Natural History Biorepository. Although these frozen tissues are of high quality, they are not live samples.

The Smithsonian Institution has also begun a long-term, comprehensive marine monitoring and collection program, the Tennenbaum Marine Observatory Network or MarineGEO, at various field sites over multiple longitudinal and latitudinal gradients. Voucher specimens—representatives of taxa that may be used as a permanent reference for examination and study—will be accessioned and catalogued at the USNM and other natural history museums. This is both exciting and challenging because, in addition to standard collections, this opportunity allows natural history museums to build large marine cryopreserved collections and maintain unique marine biodiversity materials.

These efforts–building a biorepository and monitoring marine habitats—inspired us to develop a practical, diploid cell cryopreservation protocol. Cryopreserved diploid cells make the archive a living collection that will be accessible for decades, and thus, indispensable to address a wide range of academic and societal questions. The live samples maintained, or suspended, at ultra-cold temperatures will include sperm, eggs, stem cells, endo- and ecto-bacterial cells, embryonic cells and other important cell types that include their genetic material. These living cells will also allow the propagation of new cells to increase populations, perhaps minimizing the need to recollect these same species in the future.

The broad application potential of cryopreserved material, coupled with the persistent and rapid decline of global biodiversity, presents a compelling case for the collection and maintenance of cryopreserved specimens of taxonomically and geographically diverse species. Our research focus is fishes, but we see the future of this preservation method and collection building effort across all taxa. We urge natural history museums to include the cryopreservation of spermatogonial cells to ensure the gold standard in biodiversity maintenance. By adding these novel collections, natural history museums will conserve biological materials of the highest standards and potential and provide outstanding research opportunities for many years to come.

### Article Impact Statement

A simplified protocol to cryopreserve and archive marine fish diploid spermatogonial cells to aid in conservation and phylogenetic/systematic molecular analyses.

## Electronic supplementary material

Supplementary Information
