# Supplementary material for: Cryopreservation of Fish Spermatogonial Cells: The Future of Natural History Collections
Source: Sci Rep. 2018 Apr 18;8:6149. doi: 10.1038/s41598-018-24269-3 (PMC5906666; doi:10.1038/s41598-018-24269-3)
Supplement: Supplementary file 1 — Supplementary Information [file 41598_2018_24269_MOESM1_ESM.docx]

**Cryopreservation of Fish Spermatogonial Cells: The Future of Natural History Collections**

Mary M. Hagedorn^1,2^, Jonathan P. Daly^1,2^, Virginia L. Carter^1,2^, Kathleen S. Cole^3^, Zeehan Jaafar^4,5^, Claire V. A. Lager^1,2^ and Lynne R. Parenti^5^

^1^Smithsonian Conservation Biology Institute, Front Royal VA 22360, United States of America

^2^Hawaii Institute of Marine Biology, 46-007 Lilipuna Rd, Kaneohe, HI 96744, United States of America

^3^University of Hawai‘i at Mānoa, 2538 McCarthy Mall, Edmondson Hall 216, Honolulu, HI 96822, United States of America

^4^Department of Biological Sciences, 14 Science Drive 4, National University of Singapore, Singapore 117543

^5^Department of Vertebrate Zoology, P.O. Box 37012, MRC 159, National Museum of Natural History, Smithsonian Institution, Washington, D.C. 20013-7012, United States of America

**Keywords**: biodiversity; collection protocol; conservation; molecular sequence data; vouchers

*Correspondence and requests for materials should be addressed to:

Dr. Mary Hagedorn

Smithsonian Conservation Biology Institution

Hawaii Institute of Marine Biology

46-007 Lilipuna Rd

Kaneohe, HI 96744

E-mail: [hagedornm@si.edu](mailto:hagedornm@si.edu); 808-520-1368 (TEL); 808-236-7443 (FAX)

**Supplemental Information**

**The DNA Barcode of *Asterropteryx semipunctata***

The mitochondrial cytochrome c oxidase subunit 1 (COI)--the DNA barcode[^35^](file:///C:\Users\Parentil\Desktop\Publication%20on%20Hawaii\Hagedorn%20et%20al%20092617%20FINAL.docx#_ENREF_35)—was sequenced to test our hypothesis that the populations of *A. semipunctata* that we sampled represented a single species and, if so, that the sequences matched others identified as *A. semipunctata* in GenBank. The DNA barcode was sequenced from gonadal or muscle tissue fixed in 95% ethanol from six individuals, three males and three females of *A. semipunctata*, collected from the Lanai Suites and Sandflat Fore Reef Beach sites (Table S1).

Extraction, amplification and sequencing of the DNA were performed in the Laboratories of Analytical Biology (LAB), National Museum of Natural History. Total genomic DNA was extracted from ethanol-fixed muscle or fin tissue using the Qiagen DNeasy Blood & Tissue Extraction Kit following the protocol for Purification of Total DNA from Animal Tissues (Spin-Column). Step 7 was modified as follows: 100 μl, instead of 200 μl, of Buffer AE was pipetted onto the membrane and centrifuged. This elution step was repeated to give a total elution of 200 μl. Quality of genomic DNA was tested using gel electrophoresis (1.5% agarose gel and SB Buffer). Amplification of mtCOI of the targeted fragment through the polymerase chain reaction (PCR) was performed for each sample in a total 10 μl reaction containing 3.4 μl of sterile water, 1μl of the genomic DNA, 5μl of GoTaq Mastermix, and 0.3 μl of a forward primer and 0.3 μl of a reverse primer. PCR and DNA sequencing for the teleost fish DNA barcode sequence (COI) were performed with three primer sets: Set 1: C_FishF1t1 and C_FishR1t1 (primer cocktails) (Ivanova et al. 2007), Set 2: jgLCO1490 and jgHCO2198 (Geller et al., 2013) and Set 3:FishCOILBC and FishCOIHBC (Handy et al. 2011). Negative controls were included for each primer set reaction. The PCR protocol was: 1 cycle of initial denaturation at 95°C for 5 minutes, followed by 1 cycle of denaturation at 95°C for 45 s, annealing at 50°C for 45 s, and extension at 72°C for 60 s; these steps were repeated for an additional 34 cycles; it was ended by final extension at 72°C for 5 m. PCR products were visualized using gel electrophoresis and cleaned using ExoSAP-IT (USB, Cleveland, OH). Purified products from Set 1 were sequenced using a 3730xl DNA analyzer (Applied Biosystems, Inc.). A quick sequencing quality check was performed using Geneious 7.04. The orientation of individual sequences was checked and trimmed; contig sequences were assembled and run through a BLAST search. All sequences were uploaded to GenBank (Table S1).

RESULTS

The BLAST searches for pairwise similarity of the DNA barcode sequences of six representative specimens of *A. semipunctata* recovered a match with existing mtCOI sequences of *A. semipunctata* previously uploaded to GenBank (Table S1). We thus corroborate the hypothesis that all our specimens represent a single species.

Table S1. *Asterropteryx semipunctata* collected at two localities on Coconut Island, April-May, 2013 used in the DNA Barcode (COI) analyses. A unique code number identified each *A. semipunctata* specimen and its tissue in the laboratory. All material is housed in the USNM under the listed catalog number. GenBank accession numbers for the tissues are listed under NCBI COI.

Locality Field Code USNM Sex NCBI COI

Lanai Suites 042913-1 421647 male KR059867

Lanai Suites 042913-2 421648 female KR059868

Lanai Suites 042913-7 421653 female KR059869

Sandflat Fore Reef Beach 050113-32 421678 male KR059870

Sandflat Fore Reef Beach 050113-36 421682 male KR059871

Sandflat Fore Reef Beach 050113-41 421687 female KR059872
